# An Easy Approach to Control β-Phase Formation in PFO Films for Optimized Emission Properties

**DOI:** 10.3390/molecules22020315

**Published:** 2017-02-18

**Authors:** Qi Zhang, Lang Chi, Gang Hai, Yueting Fang, Xiangchun Li, Ruidong Xia, Wei Huang, Erdan Gu

**Affiliations:** 1 Key Laboratory for Organic Electronics & Information Displays (KLOEID), Jiangsu-Singapore Joint Research Center for Organic/Bio Electronics & Information Displays, Institute of Advanced Materials (IAM), Nanjing University of Posts and Telecommunications, 9 Wenyuan Road, Nanjing 210046, China; zq890531@163.com (Q.Z.); chilang_888@163.com (L.C.); haigang686@163.com (G.H.); fangyuetingfly@163.com (Y.F.); iamxcli@163.com (X.L.); 2 Jiangsu-Singapore Joint Research Center for Organic/Bio-Electronics & Information Displays, Institute of Advanced Materials (IAM), Nanjing Tech University, 30 South Puzhu Road, Nanjing 211816, China; iamwhuang@njtech.edu.cn; 3 Institute of Photonics, University of Strathelyde, 106 Rottenrow, Glasgow, G4 0NW, UK; erdan.gu@strath.ac.uk

**Keywords:** polymer optoelectronics, poly(9,9-dioctylfluorene), β-phase, amplified spontaneous emission, organic light emitting diode

## Abstract

We demonstrate a novel approach to control β-phase content generated in poly(9,9-dioctylfluorene) (PFO) films. A very small amount of paraffin oil was used as the additive to the PFO solution in toluene. The β-phase fraction in the spin-coated PFO films can be modified from 0% to 20% simply by changing the volume percentage of paraffin oil in the mixed solution. Organic light emitting diodes (OLEDs) and amplified spontaneous emission (ASE) study confirmed low β-phase fraction promise better OLEDs device, while high β-phase fraction benefits ASE performance.

## 1. Introduction

Conjugated polymers are an important class of semiconductors, due to their low-cost, easy-fabrication, environment-friendly, and flexibility features. Compared to the inorganic opponent, these materials can be tailored through chemical structure modification or conformation control during processing. Among all the conjugated polymers, poly(9,9-dioctylfluorene) (PFO) and its derivatives are one class of the most attractive candidates for light-emitting diodes [1,2,3,4] and lasers [5,6,7,8,9,10,11] as a blue-emitter. Research on PFO was mainly focused on the luminance efficiency increase and color purity of the PFO light emitting diode (LED) and the threshold reduction of PFO laser. PFO is also widely studied because of the presence of the well-defined planar chain conformation resulting in extended conjugation length [12], which is the so-called “β-phase.” In the “β-phase” formation, the electronic delocalization is increased, characterized by red-shifted absorption and fluorescence spectra, increased vibronic structure, reduced Stokes shift [13,14,15,16,17]. Well controlled “β-phase” content in a glassy PFO film can facilitate the performance of optoelectronic devices: an organic light emitting diode (OLED) with higher luminance efficiency and better stability [18] and a laser with a lower threshold [19,20]. The very first report [15] of “β-phase” observation was carried out by cooling spin-coated PFO films on fused silica substrates to liquid nitrogen temperature (−196 °C) and slowly reheating them to room temperature. After that, many methods have been developed to generate the “β-phase” conformation in solid PFO samples: processing PFO in poor solvent (such as cyclohexane [14] or cyclopentanone [21]) or in good solvent but with a high boiling point (such as isodurene) [21]; introducing small amount of high-boiling-point additives (such as 1,8-diiodooctane [22,23,24] or polyphenylether [25]) in good solvent followed by spin-coating; exposing PFO films to good solvents in liquid or vapor form (leading the film undergoing the “swelling stress”) [26,27,28] or to saturated vapors of moderately good solvents such as THF [29], n-octane [30], chlorobenzene [28], and toluene [13,17,26,30,31] (so-called solvent vapor annealing); dipping glassy PFO films into solvent/non-solvent mixtures, such as THF–methanol [32,33] or toluene–methanol [32] for tens of seconds at a time.

In this work, we find that a desirable fraction of β-phase in PFO could be generated by introducing a certain volume of paraffin oil into PFO solution. Specifically, by changing the volume percentage of paraffin oil (from 0 to 0.5 vol %) in the mixture PFO solution, the β-phase content was precisely tailored between 0% and 20%. The investigations detailed below indicate that a small fraction of β-phase content (around 2%) can improve color purity and device performance in OLEDs, while a relatively high fraction β-phase content (>7%) is beneficial for lowering lasing threshold. Therefore, we can control the fraction of “β-phase” conformation of the PFO films to fit the different application such as OLED or lasers.

## 2. Results and Discussion

**Figure 1:**
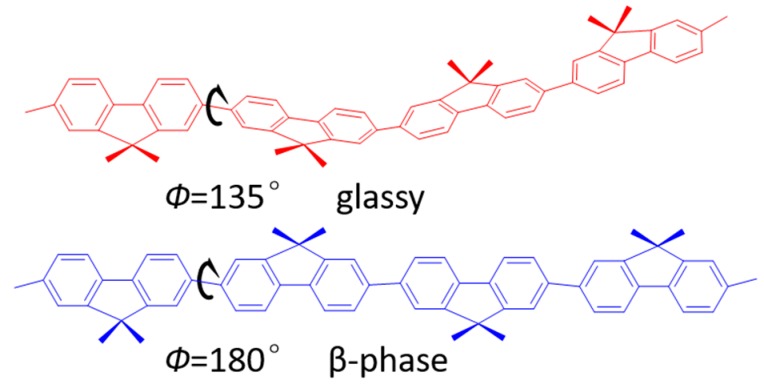
The differences inter-monomer torsion angle Ф between the glassy and β-phase PFO chain conformations. The n-octyl side-chains of PFO (C_8_H_17_) are omitted for clarity.

Figure 1 shows the conformation differences between the glassy and β-phase PFO chain. The inter-monomer torsion angle is 135° for glassy and 180° for β-phase PFO, respectively. This can be assigned to the relatively weak, typically van der Waals, interchain bonding which is already well studied.

**Figure 2:**
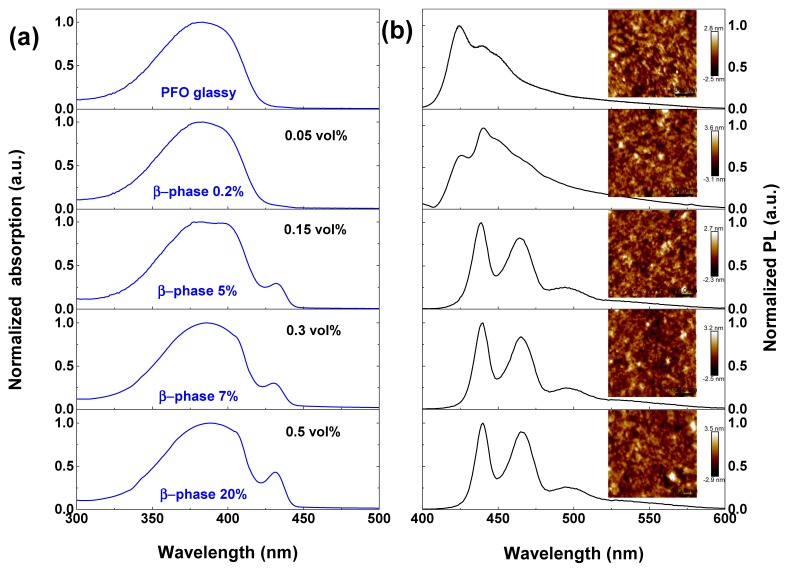
(**a**) Normalized absorption spectra and (**b**) PL spectra of the PFO films spin-coated from mixture containing different volume percentage of paraffin oil. The volume percentage of paraffin oil and the calculated β-phase fraction are labeled in the absorption spectra. AFM images of each film are shown as inset in the PL spectra.

**Figure 3:**
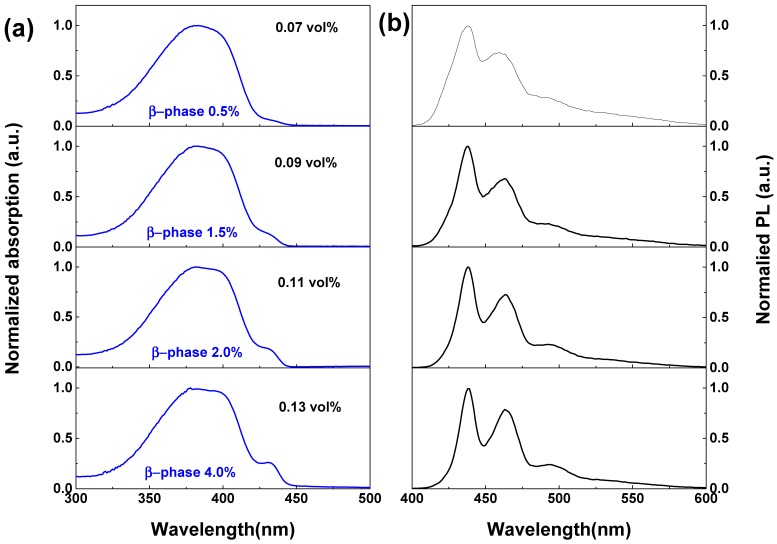
(**a**) Normalized absorption and (**b**) PL spectra of the mixed phase PFO films spin-coated from mixture containing different volume percentage paraffin oil ranging from 0.07 to 0.13 vol %.

Figure 2 shows the normalized absorption and PL spectra of the PFO films fabricated from solution mixed with a different percentage of paraffin oil from 0 to 0.5 vol %. The calculated β-phase fractions are labeled in the absorption spectra. The corresponding AFM images are shown as an inset in the PL spectra. The absorption peak of pristine PFO film is located around 383 nm. The absorption spectra of mixed-phase PFO films can be seen as a linear superposition of glassy and β-phase. The β-phase chain conformation is well-defined and distinct from the glassy conformation by a newly emerging peak at around 431 nm, which undergoes a slight redshift from 430 nm to 432 nm with increasing β-phase fraction. The fraction of β-phase was estimated from the ratio of integrated spectral areas of the β-phase and the mixed phase, taking the oscillator strength difference between phases into account (see details in Supplementary Materials) [33]. The PL spectra of the glassy and mixed phases PFO in Figure 2b shows that the S1-S0 0-0 peak (~430 nm) of the β-phase is already evident even at a β-phase fraction as low as 0.2%. The PL is completely dominated by β-phase emission at fractions >1.5% (see Figure 3). The red shift of PL spectra was also observed from the color differences between glassy and β-phase rich PFO films under 365 nm flashlight shown in Figure S1 in Supplementary Materials. Ariu et al. [13] assigned rapid energy transfer from the higher glassy matrix to the β-phase chromophores to be the primary mechanism for populating the excited singlet state manifold of the β-phase. AFM images depicted in Figure 2b show the morphology and roughness of all samples has no significant difference. The absorption and PL of films spin-coated from mixture with higher volume percentage (>1 vol %) of paraffin oil is shown in Figure S2. The mechanism for β-phase generation in such a mixture solution is inferred to be related to the high boiling point (>300 °C) and the poor solubility for PFO (PFO powder is bearly dissolved) of paraffin oil. We suppose these effects lowering the quality of the overall solvent, which slows down the kinetics of film forming during spin coating. Toluene, a good solvent for PFO, evaporates faster and leaves the bad solvent behind. During the extended “drying” procedure, mechanical stress is applied on PFO chains, which could favor the formation of β-phase as indicated in an early report [21].

To monitor the procedure glassy phase transforms to β-phase, we carried out more detailed studies on the mixed phase PFO films spin-coated from the PFO/toluene solution (15 mg/mL) mixed with 0.07 to 0.13 vol % paraffin oil. As shown in Figure 3a, the S0-S1 0-0 absorption peak of the β-phase (λ ~ 430 nm) first appears like a shoulder when the paraffin oil volume was 0.07 vol % in the mixture. By increasing the volume percentage of the paraffin oil to 0.13 vol %, the characteristic absorption peak of the β-phase at λ ~ 430 nm becomes obvious. However, the PL spectra in Figure 3b are dominated by β-phase emission; even the volume percentage of the paraffin oil is as low as 0.07 vol %. Note that the PL spectrum of the films spin-coated from the PFO/toluene solution with 0.05 vol % paraffin oil is a superposition of the glassy matrix and the β-phase emission as shown in Figure 2b. However, the rapid energy transfer from the glassy matrix to the β-phase had already completed at the 0.07 vol % paraffin oil mixture. These paraffin oil volume percentages correspond to the β-phase fraction increases from 0.3% (0.05 vol % paraffin oil) to 0.5% (0.07 vol % paraffin oil).

**Figure 4:**
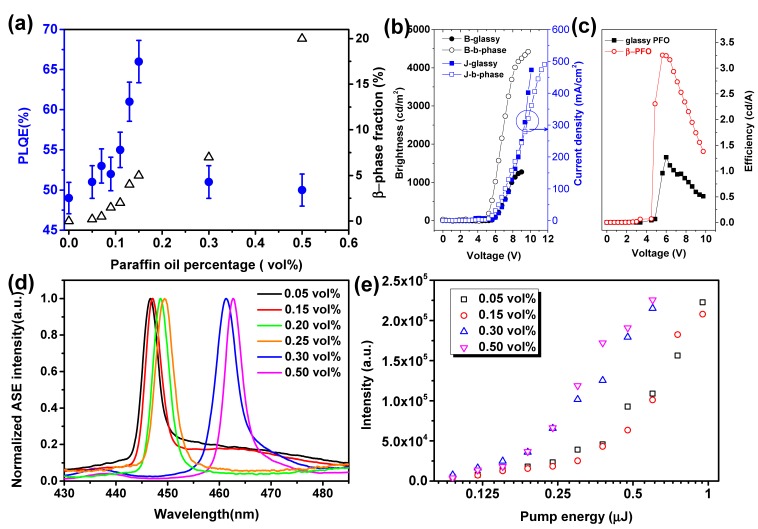
(**a**) PLQE (filled circles) and β-phase fractions (open triangles) of PFO films as a function of paraffin oil volume percentage. (**b**) J–V–B curves and (**c**) the current efficiencies as a function of the input voltage for LEDs based on pristine PFO and mixed phase PFO films (β-phase fraction 2%). (**d**) ASE spectra of mixed phases PFO films spin-coated from various mixtures with paraffin oil volume percentages: 0.05, 0.15, 0.20, 0.25, 0.30, and 0.50 vol % (β-phase fraction 0.2, 5, 5.3, 5.7, 7, 20%). (**e**) Output intensity of films made from different paraffin oil mixtures is plotted as a function of the pump energy.

After demonstrating the fraction-predictable approach to generate β-phase formation by controlling the paraffin oil volume percentage in the PFO/toluene solution, we investigated the photoluminescence quantum efficiency (PLQE) of PFO films as a function of the β-phase fraction in detail. As shown in Figure 4a, which also shows the correlation between paraffin oil volume percentage and calculated β-phase fraction (values are shown in Table S1), the PLQE of glassy PFO is 49%. With the β-phase fraction increases the PLQE of the PFO film rises continually to reach a maximum of ~66% at β-phase fraction up to 5% (paraffin oil volume percentage lower than 0.15%), then drops down to the similar PLQE value of the glassy PFO (50%) at the β-phase fraction increased to 7% (paraffin oil volume percentage 0.3%) and remains constant with the β-phase fraction further increased to 20% (paraffin oil volume percentage lower than 0.5%). These results confirm that the high PLQE could be achieved under desirable β-phase fractions by controlling the paraffin oil volume percentage.

Furthermore, we have investigated the performance of light emitting diodes (LEDs) and amplified spontaneous emission (ASE) using mixed phases PFO films as active polymer. Figure 4b shows current density–voltage–brightness (J–V–B) curves of devices based on pristine PFO and mixed phase PFO films (β-phase fraction 2%). The OLED devices are constructed as follows: ITO/PEDOT(25 nm)/polymer(70 nm)/LiF(1 nm)/Al(150 nm) (see Figure S3). Figure 4c illustrates the dependence of their corresponding current efficiencies on voltage. For pristine PFO, light turn-on voltage (at a measurable brightness of 2 cdm^−2^), maximum brightness, and current efficiency are 5.25 V, 1274 cdm^−2^ (9 V), and 1.27 cd A^−1^ (6 V and 86 cd m^−2^), respectively. For PFO with 2% β-phase, the corresponding results are 4.5 V, 4420 cdm^−2^ (9.75 V), and 3.25 cd A^−1^ (5.62 V and 527 cdm^−2^). The device using PFO film with 2% β-phase shows better performance which can be assigned to more balanced charge fluxes and more efficient charge recombination due to the efficient energy transfer and charge trapping [18].

The typical ASE spectra of the different mixed phases PFO film is shown in Figure 4d. For the film sample made from the mixture containing low paraffin oil volume percentage, therefore, low β-phase fraction, the peak position located at 446.6 (for 0.05 vol % paraffin oil, 0.2% β-phase) and 447.1 nm (for 0.15 vol % paraffin oil, 5% β-phase). As the paraffin oil volume increases the ASE peak red shifts more than 10 nm to the 461.3 nm for 0.30 vol % paraffin oil, 7% β-phase, and 462.6 nm for 0.50 vol % paraffin oil, 20% β-phase. The output intensity of the films made from different mixture is plotted as a function of pump energy in Figure 4e. The thresholds of mixed-phases PFO films are estimated from Figure 4e: when the β-phase fraction is relative low (<5%), the threshold is 325 nJ and 302 nJ for films with 0.2% and 5% β-phase. Glassy PFO content still dominate the ASE as shown in Figure 4d. When the β-phase fraction rises to 7%, the ASE thresholds decrease to 188 nJ and 187 nJ for films with 7% and 20% β-phase, respectively, almost down to the half of the ASE threshold value of the glassy content dominated films. Note that this threshold reduction point is also combined with an ASE peak jumping from 448 nm to 461.3 nm as shown in Figure 4d, confirming that the β-phase emission has dominated the ASE. The significant decrease of the ASE threshold suggests that the higher β-phase fraction is beneficial to lowering ASE threshold.

## 3. Materials and Methods

PFO (MW ~ 60000) used in this work was bought from the Xian P-led ltd. (Xi’an, China). The paraffin oil is a commercial product bought from Edwards, named “ultragrade 19.” The PFO solution was prepared as below: paraffin oil is first dissolved in toluene to make a precursory mixture (volume concentration from 0 to 50 µL/mL), and 10 µL of precursory mixture is added into 90 µL of PFO toluene solution (PFO 15 mg/mL), forming the mixture with different paraffin oil volume percentages from 0 to 0.5 vol %. All thin films were spin-coated at a speed of 2500 rpm, resulting in a thickness range of around 95 nm, followed by annealing for 5 min under 70 °C. UV-Vis absorption and PL spectra were recorded using a Shimadzu UV-3150 and RF-5300PC spectrometer, respectively. The PLQE of the films was measured by Edinburgh FLSP920 (black box system, Edinburgh, UK) with an integrated sphere. For ASE and laser measurements, samples were optically pumped witha Q-switched, neodymium ion-doped yttrium aluminum garnate [Nd^3+^:YAG] laser-pumped, type-II β-BaB_2_O_4_ [BBO] optical parametric oscillator, which delivered 5 ns pulses at a repetition rate of 10 Hz. Calibrated neutral density filters were inserted into the beam path to adjust pulse energy incident on the sample. In ASE measurements, an adjustable slit and a cylindrical lens were combined to create a narrow excitation strip vertically placed at the edge of sample film. The edge emission from samples was collected with a fiber-coupled grating spectrometer equipped with a CCD detector. At sufficient excitation intensities, the spontaneously emitted photons that were waveguided along the stripe-shaped gain region are amplified via stimulated emission. This process results in most of the light being emitted from the ends of the stripe. The pulse energy of pump light was determined by an energy and power meter. All thickness measurements were taken by using the profilemeter Dektak XT (Bruker co., Beijing, China).

## 4. Conclusions

We demonstrate a simple approach to precisely control β-phase fraction in PFO film. Various β-phase fractions from 0.2% to 20% have been obtained by adding paraffin oil into PFO/toluene solution at volume percentage of 0.05–0.5 vol %. The paraffin oil we used as an additive is a common commercial product. No special treatment is need prior to or after deposition of the films, which provides great convenience for device fabrication. The emission-related investigation shows that the films with lower β-phase fraction (less than 5%) exhibit higher PLQE (up to 66%) and better OLED performance, while those films with higher β-phase fraction (7%–20%) show lower threshold in ASE tests. This study shows it is possible to control the β-phase generation in the mixed phase PFO films to fulfill different applications such as OLEDs or lasers.

## Supplementary Materials

The following are available online at http://www.mdpi.com/1420-3049/22/2/315/s1. Figure S1: Images of glassy PFO films and PFO film with various β-phase fractions when exposed under 365 nm flashlight; Figure S2: Normalized absorption and PL of the film spin-coated from mixed PFO solution with 1, 2, and 3 vol % paraffin oil; Figure S3: OLED device structure; Table S1: Values of the PLQE and β-phase fraction of PFO films spin-coated from solutions with different paraffin oil volume percentage.
