# Supplementary material for: An Easy Approach to Control β-Phase Formation in PFO Films for Optimized Emission Properties
Source: Molecules. 2017 Feb 18;22(2):315. doi: 10.3390/molecules22020315 (PMC6155933; doi:10.3390/molecules22020315)
Supplement: Supplementary file 1 [file molecules-22-00315-s001.pdf]

# Supplementary Materials: An Easy Approach to Control β-Phase Formation in PFO Films for Optimized Emission Properties

**Qi Zhang, Lang Chi, Gang Hai, Yueting Fang, Xiangchun Li, Ruidong Xia, Wei Huang and Erdan Gu**

## Calculation of β-phase Fraction

The absorption spectra of mixed-phase PFO films are understood to comprise a linear superposition of glassy and β-phase contributions. To estimate the fraction of β-phase chain segments (hereafter simply referred to as "β-phase fraction") in a PFO thin film, the absorption spectrum of the β-phase chain segments was extracted by normalizing the corresponding mixed phase and reference glassy absorption spectra at ~345 nm, followed by subtraction. The β-phase fraction is estimated from the ratio of integrated spectral areas of the β-phase, $A_\beta$, and the mixed-phase, $A_{mixed}$, (where $A_{mixed} = A_{glassy} + A_\beta$) absorption spectra, taking into account that the relative difference in oscillator strength, $f_{osc}$, used time-dependent density functional theory (DFT) to estimate $f_{osc}^{\beta} = 1.08 \times f_{osc}^{glassy}$. Consequently, the β-phase fractions in PFO films were calculated by Equation (1):

$$\beta \text{ phase fraction} = \frac{A_\beta}{A_{mixed}} = \frac{A_\beta}{A_\beta + \left((A_{mixed} - A_\beta) \times 1.08\right)} \quad (1)$$

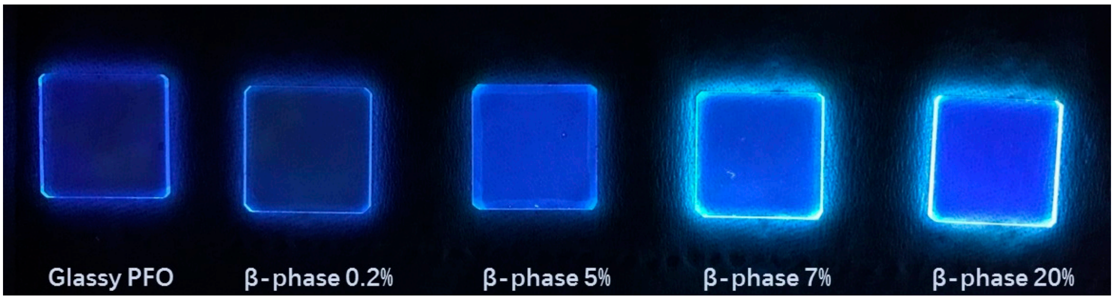


**Figure S1**. Images of glassy PFO films and PFO film with various β-phase fractions when exposed under 365 nm flashlight.

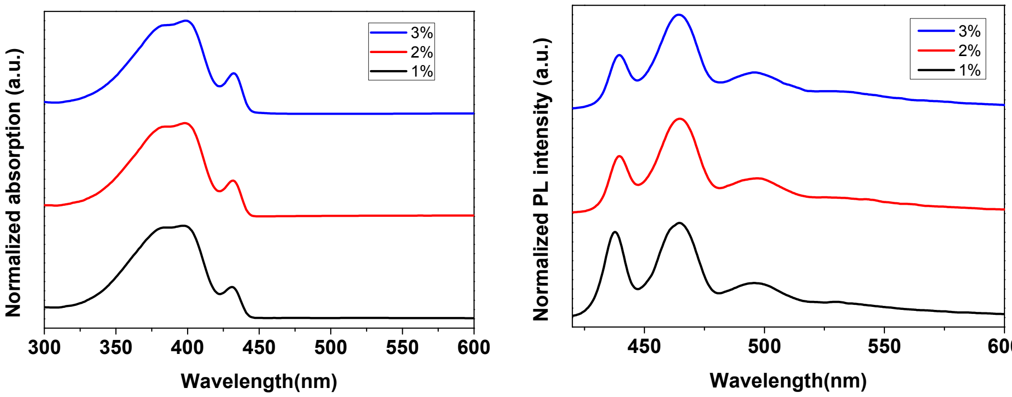


**Figure S2.** Normalized absorption and PL of the films spin-coated from mixed PFO solution with 1, 2 and 3 vol % paraffin oil.

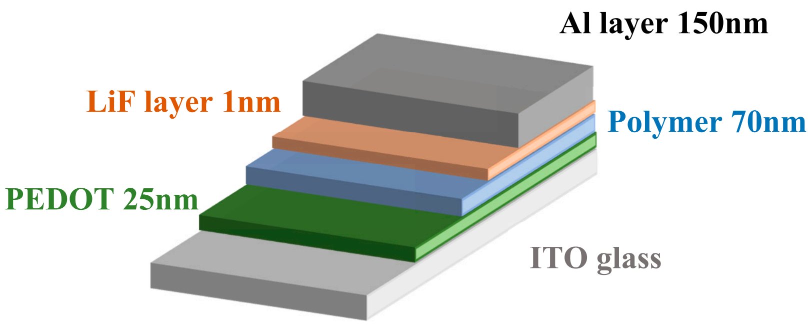


**Figure S3.** OLED device structure.

**Table S1**. Values of the PLQE (Photoluminescence quantum efficiency) and β-phase fraction of PFO film samples made from different solution with paraffin oil volume percentages.

| Paraffin Oil Volume Percentage (vol%) | β-Phase Fraction (%) | PLQE (%) |
|---|---|---|
| 0 | 0 | 49 |
| 0.05 | 0.2 | 51 |
| 0.07 | 0.5 | 53 |
| 0.09 | 1.5 | 52 |
| 0.11 | 2 | 55 |
| 0.13 | 4 | 61 |
| 0.15 | 5 | 66 |
| 0.30 | 7 | 51 |
| 0.50 | 20 | 50 |
